# Pharmacokinetics of Doxycycline in Plasma and Milk after Intravenous and Intramuscular Administration in Dairy Goats

**DOI:** 10.3390/ani14162416

**Published:** 2024-08-20

**Authors:** José Martínez, Elisa Escudero, Elena Badillo, María Teresa Yuste, Juan Sebastián Galecio, Pedro Marin

**Affiliations:** 1 Department of Pharmacology, Faculty of Veterinary Medicine, University of Murcia, 30100 Murcia, Spain; jose.martinez10@um.es (J.M.); escudero@um.es (E.E.); pmarin@um.es (P.M.); 2 Escuela de Medicina Veterinaria, Colegio de Ciencias de la Salud, Universidad San Francisco de Quito, Quito EC 170157, Cumbayá, Ecuador; jgalecio@usfq.edu.ec

**Keywords:** bioavailability, dairy goats, doxycycline, milk, pharmacokinetics

## Abstract

**Simple Summary:**

Doxycycline is a tetracycline antibiotic used to treat bacterial infections in many species. Prior to the use of any antibacterial agent in veterinary medicine, pharmacokinetic studies in the target species are required to determine an optimal dosing regimen to prevent the selection of resistant bacteria and to ensure therapeutic success. To date, information on the pharmacokinetics of doxycycline in lactating goats is very scarce. Therefore, the aim of this study was to evaluate the disposition kinetics and the excretion in milk of doxycycline after intravenous and intramuscular administration in dairy goats. Volumes of distribution were found to be medium, which suggests a moderate distribution of this antibiotic in tissues. After intramuscular administration, all goats developed lameness, which resolved 24–48 h post-administration. The bioavailability of doxycycline after IM injection was relatively low. Doxycycline rapidly crossed the blood–milk barrier, but exposure to the antimicrobial and the concentrations reached in milk were lower than those obtained in plasma. Therefore, doxycycline IM could be useful for infections by highly susceptible bacteria in the mammary gland of goats.

**Abstract:**

Doxycycline is a second-generation tetracycline, marketed in different species for treating infections caused by susceptible bacteria. Little information is available on the pharmacokinetics of doxycycline in lactating goats. The objective of this study was to establish the disposition kinetics of doxycycline after parenteral administration (intravenous and intramuscular) in dairy goats and its elimination in milk. A cross-over model was designed (*n* = 6). Doxycycline was dosed at 5 mg/kg for intravenous administration and 20 mg/kg for extravascular administrations. Noncompartmental pharmacokinetic methods were used to calculate plasma concentration–time data. The V_z_ value suggests a moderate distribution of this antibiotic in goats, with a value of 0.85 L/kg. A low bioavailability (F = 45.60%) of doxycycline following an intramuscular injection was observed, with all animals exhibiting signs of lameness. Doxycycline rapidly crossed the blood–milk barrier, but exposure to the antimicrobial and the concentrations reached in milk were lower than those obtained in plasma. Although PK/PD ratios may be low with the pharmacokinetic data obtained with this formulation of doxycycline, at this dose and route of administration, doxycycline after IM administration could be useful for infections by moderate or highly susceptible bacteria in the mammary gland of goats. However, it may be necessary to test different doses of doxycycline or other routes of administration to achieve better surrogate markers and to establish repeated dosing regimens and clinical efficacy.

## 1. Introduction

The European Union has more than 11 million heads of goats. Greece leads the EU with 2.9 million heads, followed by Spain (2.4 million), Romania (1.5 million), France (1.3 million) and Italy (1.0 million). Unlike the sheep sector, the goat sector is mainly oriented towards dairy production [1]. Currently, 15% of the world’s goats’ milk is produced in the countries of the European Union. Spain is the second largest producer of goats’ milk in Europe [2]. Although there is a growing interest in consuming dairy goat products, there are still few medicines approved to treat these animals. Moreover, the body of research investigating the pharmacokinetics of various drugs in this species continues to expand. However, goats’ milk is generally richer in fat and protein than bovine milk and this difference in the concentration of the components may lead to changes in the disposition of the drugs between the species [3].

Contagious agalactia (CA) is a severe multi-etiological disease that has an important economic impact in countries with sheep and goat dairy industries, especially in Mediterranean countries, where the disease is considered endemic. Despite the term “agalactia”, this pathology does not exclusively affect milk production, nor does it only appear in lactating females or in mammary tissues. Clinical signs of the disease are diverse and include mastitis, keratoconjunctivitis, arthritis, pneumonia and septicaemia [4,5,6]. Because of the prevalence of the disease and the serious economic losses it causes, it is listed by the World Organisation for Animal Health as a notifiable disease, and it is considered one of the costliest small ruminant production diseases. Four pathogens, namely *Mycoplasma mycoides* subspecies *capri*, *M. agalactiae*, *M. putrefaciens* and *M. capricolum* subspecies *capricolum*, are responsible for CA. These pathogens are excreted in a range of secretions and fluids, including milk, ocular, nasal, pulmonary, vaginal, auricular, semen, synovia and blood. Although the main agent is *M. agalactiae* in small ruminants, all four bacterial species contribute significantly to economic losses. In goats, several mycoplasma species may be present in the same herd or individually, causing mixed infections that, together with the presence of other mycoplasmas, which are considered to be non-pathogenic, further complicate the diagnosis and implementation of control measures [7,8,9]. Antimicrobial treatment, vaccination and good animal husbandry are the main methods to control CA, but clinical relapses are frequent because of the long-term commitment required by farmers [4]. The pharmacological treatment of CA may be undertaken with the use of a variety of antimicrobial agents. However, the emergence of diverse resistance mechanisms within *Mycoplasma* spp. to combat the antimicrobial action of these drugs, coupled with the paucity of medicaments specifically formulated for use in small ruminants, renders their application challenging [10].

Doxycycline is a second-generation tetracycline, derived from oxytetracycline, characterised by higher lipid solubility than the first-generation tetracyclines. Currently, this antibiotic is commercially available as calcium salt, hyclate salt and the monohydrate salt. In veterinary medicine, the hyclate salt is the most commonly used form, as its solubility in water is much better than the monohydrate form [11,12,13]. Doxycyclina hyclate is available as an injectable solution, water-soluble or lactodispersable powder, and tablets and capsules. This antibiotic is authorized by the European Medicine Agency (EMA) in different species for the treatment of infections of the respiratory tract, urinary tract and intestines caused by susceptible microorganisms [14]. To date, there is no EMA-approved doxycycline drug in goats.

In bacteria, doxycycline inhibits protein synthesis by binding reversibly to the 30S unit of the bacterial ribosome. This antimicrobial has a broad spectrum of activity against a wide range of microorganisms. These include anaerobic and aerobic Gram-negative and Gram-positive bacteria and intracellular pathogens such as *Rickettsia* spp., *Chlamydia* spp. and some *Mycoplasma* spp. [15,16,17,18,19]. In addition, various anti-inflammatory and antineoplastic functions have been attributed to doxycycline. Because of this broad antibacterial spectrum and anti-inflammatory function, doxycycline has been widely used in numerous domestic animals. Therefore, to avoid the selection of resistant bacteria due to their widespread use, the pharmacodynamic and pharmacokinetic properties of this drug should be taken into account when applying the principles of prudent use [13]. The kinetic dispositions of doxycycline have been studied in various livestock species such as pigs, horses, donkeys, calves, sheep and goats [20,21,22,23,24,25,26,27,28,29]. In the latter species, the pharmacokinetics of this antibiotic have been studied after intravenous, intramuscular and subcutaneous administration, but its elimination in milk has only been studied after IV administration [30].

Another important aspect for the use of a drug is its safety profile. Doxycycline has a broad therapeutic index and is relatively well tolerated by most animal species, although it is considered an irritant drug. Oral administration may cause various digestive adverse effects such as irritation of the stomach and oesophagus, with risk of ulcerations and vomits. Therapeutic doxycycline administrations in dogs caused anorexia, diarrhoea, vomiting and liver damage in some animals of this species [31]. An intravenous injection of doxycycline caused hypertension, tachycardia and even death in horses, while, in sheep and goats, it caused sialism, tachypnoea, tremors and limb weakness. In addition, after intramuscular administration of this antibiotic, symptoms of pain such as screaming, restlessness, lying down and swelling at the injection site have been reported in different species [27,28,32].

Therefore, based on the limited information of doxycycline pharmacokinetics in lactating goats, the objective of this investigation was to establish the disposition kinetics and milk excretion of doxycycline after intravenous (IV) and intramuscular (IM) administration in dairy goats.

## 2. Materials and Methods

### 2.1. Animals

The trial involved six lactating *Murciano-Granadina* goats (Veterinary Teaching Farm, University of Murcia, Murcia, Spain) with a weight of 43.25 ± 3.98 kg and an age range of 3.08 ± 0.80 years. For a period of at least 21 days prior to the investigation, the goats were fed a diet that was free of antibiotics. Animal health was assessed by physical examination. Before and after doxycycline injection, general health parameters of the goats were assessed at different times (1, 10, 24, 24, 48 and 72 h). The Bioethics Committee of our institution has approved the research protocol (CEEA 758/2021).

### 2.2. Experimental Design

A cross-over study has been developed in two periods of time, with a wash-out period of at least 15 days between the periods. Each goat randomly received a single IV and IM injection of doxycycline at a dose of 5 mg/kg for IV administrations (Vibravenosa 100 mg solution for IV injection, HOSPIRA INVICTA, Madrid, Spain) or 20 mg/kg for IM administrations (DFV Doxivet Injectable, DIVASA-FARMAVIC, Barcelona, Spain). For intravenous administration, the drug was administered into the left jugular vein, and IM injections were made into the semimembranosus muscle (injection volume range 8–9 mL, depending on the exact weight of each animal). To avoid adverse events, doxycycline was administered intravenously slowly (over 1 min) as a bolus and at a lower dose (5 mg/kg) than that used for IM administration. Blood was collected from the right jugular vein into heparinised containers at 0 (pre-treatment), 0.083 (only after IV administration), 0.167, 0.25, 0.5, 0.75, 1, 1.5, 2, 4, 6, 8, 12, 24, 48, 72 and 96 h after administration and centrifuged at 1500× *g* for 10 min. Plasma was collected and stored at −40 °C until analysis. Milk samples were obtained before and at 1, 2, 4, 8, 12, 24, 48 and 96 h post-administration after complete evacuation of the udder by manual milking. For each sampling time, milk was collected in a tank for each goat and the volume of milk obtained was recorded. Individual milk samples were taken directly from these containers to determine milk concentrations of doxycycline, transferring 2 mL into duplicate Eppendorf tubes (stored at −40 °C). To assess damage at the site of administration, changes in skin temperature, lameness, swelling in the vein and the presence or absence of pain at the injection sites were observed.

### 2.3. Analytical Methods

Concentrations of doxycycline in milk and plasma were determined using an HPLC assay with a fluorescence detector. The HPLC equipment (Shimadzu, Tokyo, Japan) used was the same as that used for the quantification of other drugs in our lab [33]. Doxycycline and internal standard (danofloxacin) were bought from Cymit Química (Barcelona, Spain).

To 200 µL of plasma or milk, 10 µL of IS solution (10 µg/mL) was added. Plasma proteins were precipitated by adding a mixture of 100 µL of methanol and 100 µL of a 1:2 solution of trifluoroacetic acid and methanol. This sample was then vortexed for 10 s and sonicated for 5 min. The sample was centrifuged for 10 min at 14,000 rpm. The supernatant was injected into the HPLC system at a rate of 50 µL per sample. An XBRIDGE, C18 column (100 mm, 4.6 mm, 3.5 µm) supplied by WATERS CROMATOGRAFÍA (Barcelona, Spain) was used. The mobile phase was composed of (A) an aqueous phase containing 50 mM ammonium acetate, 50 mM magnesium chloride and 1 mM Na_2_EDTA, buffered to pH 7.5 with ammonium hydroxide. Finally, 1 mL of triethylamine was added to each 500 mL of mobile phase A; (B) acetonitrile. The isocratic method employed a 15:85 volume ratio of aqueous phase A and phase B. The flow rate was 1 mL/min. Detection was made at λ_excitation_ = 380 nm and λ_emission_ = 520 at 20 °C. The total duration of the analysis was 12 min.

### 2.4. Method Validation

The method was validated according to the FDA Guidance for the Validation of Bioanalytical Methods [34]. The parameters assessed were as follows: accuracy, precision, linearity, lower limit of detection (LOD), lower limit of quantification (LOQ), recovery, selectivity and carryover. The complete protocols followed to validate each of the above parameters, as well as the coefficients of variation that were considered acceptable, were described previously [33]. Seven concentrations of doxycycline plus IS in plasma or milk samples were analysed to determine the linearity of the proposed chromatographic method. Three replicates of each level were analysed. The concentration giving a signal-to-noise ratio ≥ 3 was used as the lower limit of detection for doxycycline. The lowest concentration of the calibration curve with a %CV accuracy of less than 20% was the limit of quantitation. Five replicates of plasma or milk samples from four quality controls spiked with IS were analysed to calculate the precision and accuracy of the method (intraday: five replicates of each concentration were analysed on the same day; interday: five replicates of each concentration were analysed on three consecutive days). Three concentrations were analysed in the recovery tests (five samples at each concentration level were analysed).

### 2.5. Pharmacokinetic Analysis

Noncompartmental parameters were calculated using the WinNonlinTM (WinNonlin; Pharsight Corporation; Mountain View, CA, USA). Data below the LOQ were discarded. The abbreviations and descriptions of each pharmacokinetic parameter can be found in the footnote of Table 1 and Table 2.

### 2.6. Statistical Analysis

Statistical analysis was performed using Solid version 2.3.28 of the Jamovi software [35]. With the exception of half-lives, which were calculated using the harmonic mean, the remaining pharmacokinetic data were determined using the arithmetic mean and standard deviation. Normality was tested using the Shapiro–Wilk test. A paired *t*-test was employed to assess the statistical differences between data sets when the data were normally distributed. Conversely, a Wilcoxon signed rank sum test was utilised when the data were not normally distributed. Differences were considered significant if *p* < 0.05.

## 3. Results

### 3.1. Animals

After administrations of doxycycline at different doses, goats did not show any systemic adverse effects such as diarrhoea or high fever. No signs of discomfort or inflammation were observed at the injection sites after IV administration. Following IM administration, all goats developed lameness, which was resolved 24–48 h after antibiotic administration. The assessment was conducted by measuring the swelling in the vein, noting any changes in skin temperature and evaluating the presence or absence of pain at the injection sites.

### 3.2. Analytical Method

Peaks were obtained at 8.0 min and 5.5 min corresponding to doxycycline and IS, respectively, in goat plasma and milk. The range of concentrations of the calibration curves was linear (0.1 µg/mL to 2.5 µg/mL) in goat plasma and milk. Precision; accuracy; linear regression equations; regression coefficients and recovery for milk and plasma are presented in Table 1.

LOD (0.065 µg/mL) and LOQ (0.1 µg/mL) obtained the same values in both matrices. Finally, there were no carry-over effects. These results suggest that our HPLC method could be suitable for the quantification of doxycycline in milk and plasma from goats.

### 3.3. Pharmacokinetics

For pharmacokinetic analysis, data below the LOQ were discarded. After IV administration, in some animals, two plasma sampling points were discarded corresponding to 24 and 48 h. In milk, up to three sampling points were discarded depending on the animal corresponding to 12, 24 and 48 h. After IM administration, all plasma sampling points were included and, in milk, only one or none were discarded depending on animal and corresponding to 72 h.

**Figure 1:**
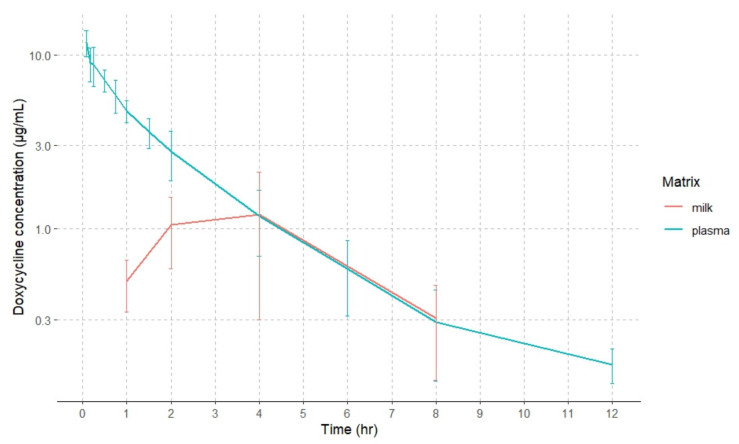
Semilogarithmic plots of plasma and milk doxycycline concentrations in goats after intravenous administration. Values are arithmetic mean ± SD (*n* = 6).

Plasma and milk concentration–time curves of doxycycline (mean ± SD) following intravenous (5 mg/kg) administration at a single dose to goats are shown in Figure 1. Doxycycline concentrations were detected up to 12 h in plasma and up to 8 h in milk after this route of administration.

**Figure 2:**
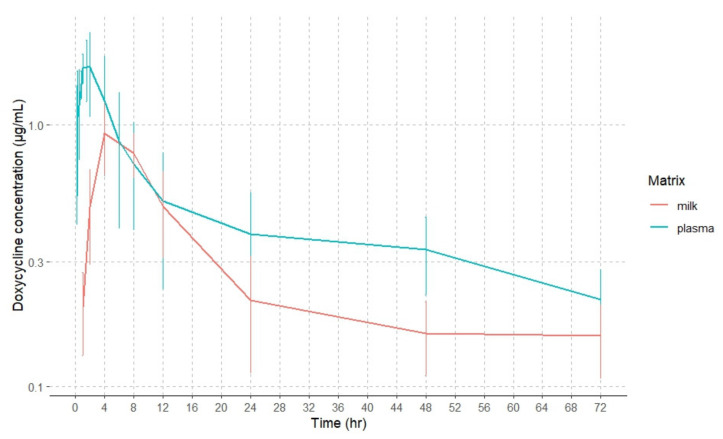
Semilogarithmic plots plasma and milk doxycycline concentrations in goats after intramuscular administration. Values are arithmetic mean ± SD (*n* = 6).

Plasma and milk concentration–time curves of doxycycline (mean ± SD) following intramuscular (20 mg/kg) administration at a single dose to goats are shown in Figure 2. Doxycycline concentrations were detected up to 72 h in plasma and milk after this route of administration. A second absorption peak can be observed in plasma concentrations after the extravascular administrations, which may be due to enterohepatic circulation processes, the flip-flop model or a slow release for the IM injection site.

Pharmacokinetic parameters of doxycycline in milk and plasma after IV and IM administrations are presented in Table 2 and Table 3, respectively. In plasma, significant differences (*p* < 0.05) between the IV administration and the IM administrations were found in λ_z_, t_½λz_, AUC_0→∞_, AUC_last_, %AUC_extrap_ and MRT.

In milk, significant differences between both administrations were found in AUC_0→∞_, AUC_last_, %AUC_extrap_, MRT, λ_z_, t_½λz_ and the two ratios.

## 4. Discussion

After IV administration of doxycycline, the half-life was 2.14 h. This value is lower than that obtained in other studies with doxycycline hydrochloride (t_½λz_ = 4.62 and 4.11 h) [25,26] and doxycycline hyclate (t_½λz_ = 4.39 h) [27] in nonlactating goats. Similar results were obtained with MRT. Therefore, if comparisons are made between dairy and non-dairy goats, doxycycline remained in plasma for much less time in lactating goats, suggesting that milk may be an important pathway for the elimination of doxycycline from the body but, also, these differences could also be due to a difference in the sensitivity of the analytical method compared with the other studies or to the use of a different breed. After IM administration, the half-life of doxycycline was longer than after intravenous administration due to the absorption phase. MRT values consistently follow the same scheme. MAT values after extravascular administration were much longer than MRT after IV treatment. This fact suggests that doxycycline follows a flip-flop model where absorption is the limiting step for doxycycline elimination from plasmas. Several studies using long-acting doxycycline formulations in calves [24] and goats [27] or a commercial oxytetracycline formulation in sheep [36] showed that absorption is often the rate-limiting step in overall tetracycline disposition and elimination after extravascular administrations.

The V_ss_ value was 0.62 L/kg, suggesting a moderate distribution of this antibiotic in goats. The value obtained in another study with doxycycline hyclate was higher (V_ss_ = 1.15 L/kg) [27]. Moreover, the obtained values with doxycycline hydrochloride were also higher [25,26]. The reason for this lower tissue distribution may be due to the fact that doxycycline has higher binding to plasma proteins than other tetracyclines [12] and, in the case of doxycycline hyclate, being more water-soluble, it has a lower capacity to cross biological membranes and a lower affinity for accumulating in adipose tissue.

The maximum concentration achieved after IM administration (C_max_ = 1.99 µg/mL) was lower than that obtained in other studies with doxycycline hydrochloride at much lower doses [25,26] and doxycycline hyclate at the same dose [27] in nonlactating goats. This suggests a rapid elimination of doxycycline through milk by an ionic trapping mechanism or chelating properties of tetracyclines. However, doxycycline absorption was relatively rapid and plasma concentration reached a maximum at 1.99 h after dosing. Similar results are found in other studies carried out in goats (t_max_ between 1–2 h) [25,26,27]. The bioavailability of doxycycline after IM injection was low, with a mean value of 45.60%. Low bioavailability for this antibiotic has also been reported after oral administration in ewes (36%) [28], goats (31%) [27] and pigs (21%) [37] and after IM administration of doxycycline hyclate in goats (51%) [27]. However, the bioavailability obtained in goats after IM administration of doxycycline hydrochloride was almost complete (F = 99.4% [25] and F = 98.99% [26]). These values indicate excellent absorption of doxycycline hydrochloride compared to doxycycline hyclate, probably due to their differences in liposolubility. Doxycycline hyclate was used in this study because this formulation appears to have the advantage that it can be formulated at higher concentrations [11,12,13]. Moreover, in our study, following IM injection, lameness due to swelling has been reported in all animals. Differences in the degree of irritation due to dose differences or different formulations may explain the low extravascular bioavailability. A possible exception may be found in the long-acting doxycycline formulation with poloxamer β-cyclodextrin matrix, which has been the subject of proposals for use in veterinary medicine. With these formulations, bioavailability was much higher after SC administration of this antibiotic in dogs (199%) [38], calves (545%) [24] and pigs (70%) [20].

A limitation of the study is the extrapolated %AUC. After IM administration, this percentage was >20% (Table 1 and Table 2). As this value is higher than 20%, the total AUC may not be reliable, indicating that more sampling is needed for an accurate estimation of the elimination rate constant and AUC. With a population pharmacokinetic analysis, this could have been handled more easily.

Doxycycline is authorized by the European Medicine Agency (EMA) in different species for the treatment of infections of the respiratory tract, urinary tract and intestines caused by susceptible micro-organisms [14]. Although it can be used in goats by extra labelling prescription, its use in dairy animals is prohibited. There are few studies on the pharmacokinetics of doxycycline in milk in different livestock species. Moreover, these studies are very old and use microbiological methods for the quantification of this antimicrobial [30,39]. The present study showed that doxycycline crosses the blood–milk barrier rapidly, as the concentrations of this antimicrobial were detected from the first sampling (at 1 h); this is consistent with the low percentage of plasma protein binding for doxycycline (33%) described previously in goats [25]. However, exposure to the antimicrobial and the concentrations achieved in milk were lower than those obtained in plasma, as indicated by the AUC_milk_/AUC_plasma_ and C_max-milk_/C_max-plasma_ ratios of doxycycline after IM administration (AUC_milk_/AUC_plasma_ = 0.51; C_max-milk_/C_max-plasma_ = 0.49). Other studies demonstrated a rapid and substantial transfer between milk and blood in dairy ruminants (cows and sheep) following intravenous administration of 20 mg/kg doxycycline. These differences may be due to a difference in plasma protein binding in the different species studied, to the different composition of goat, sheep and cow milk, and to the doxycycline salt studied. Measurable concentrations appeared at 30 min and, over time, matched those in blood, obtaining higher ratios (AUC_milk_/AUC_serum_ = 1.05) than those obtained in our study [39]. Effective systemic treatment of mastitis requires within different factors a high degree of penetration of the drugs from the blood into the milk. With the pharmacokinetic parameters obtained in milk (C_max_ = 0.95 µg/mL and AUC_last_ = 17.61 µg·h/mL), this antimicrobial would be useful intramuscularly for the treatment of clinical mastitis or elimination of carriers of CA or other bacterial infections of the mammary gland in lactating goats against strains moderately or highly susceptible to this antibiotic. Doxycycline is a bacteriostatic antibiotic and its effectiveness depends on the time between doses during which its concentration at the site of action is higher than the MIC (T > MIC) [28]. However, a number of publications have highlighted the AUC/MIC index as the most important predictor of the effect of tetracycline therapy [40,41,42]. The AUC_0–24_/MIC ratios for achieving bacteriostatic and bactericidal activity of doxycycline against *Haemophilus parasuis* in pigs were determined to be 59 and 98, respectively [43]. Minimal inhibitory concentrations of doxycycline against goat bacterial pathogens are very scarce. For example, the MIC value has been established for goat *Mycoplasma agalactiae* (MIC_90_ = 0.8 µg/mL) [44]. With this MIC value and the AUC value obtained after IM administration of doxycycline in milk, the AUC/MIC ratio is less than 59 (≈28); the MIC_90_ value for goat *P. multocida* has been reported to be 0.4 µg/mL [45]. In the case of plasma concentrations, with the afore-mentioned MIC value and the plasma AUC obtained after IM administration of doxycycline, the AUC/MIC ratio is greater than 59. However, as no killing curves have been defined for this antibiotic against *M. agalactiae* or *P. multocida*, nor other factors such as free unbound concentration of doxycycline in goat plasma or milk, efficacy cannot be predicted with certainty because the exact concentrations required to achieve a bacteriostatic effect are not known. Nevertheless, the clinical implications of these ratios must be interpreted with caution, as the therapeutic effect of antimicrobials depends on a complex set of variables. In both examples, the worst-case scenario was chosen with the MIC90 data. The primary objective of antimicrobial susceptibility testing in veterinary medicine is to select the most appropriate antibiotic for the welfare of the animal under treatment. A PK/PD cut-off differs from a PK/PD breakpoint as understood by EUCAST in that a PK/PD cut-off is derived only from PK data without clinical considerations. It is challenging to differentiate between curable and non-curable sub-populations based solely on a minimum inhibitory concentration (MIC), particularly when a specific trial has not been designed with this objective in mind. This is due to the fact that the antimicrobial susceptibility test (AST) provides insight solely into the in vitro interaction between the pathogen and the drug. It does not account for factors such as disease severity and pathogen load in the biophase, the impact of the medium components (plasma vs. milk), the immunological response to the disease, the secondary mechanisms of action of antimicrobials, or the multitude of additional clinical elements (such as the influence of concomitant drugs, the timing of treatment initiation in relation to the development of the disease and the individual disposition of drugs) that may impact the outcome of a treatment plan. Moreover, in veterinary medicine, we have more variables to consider, different species, formulations and so on. Because of this, the Probability of Target Achievement is a tool needed to define PK/PD breakpoints in animal species [46]. Therefore, it will be useful to have the data of studies exploring AUC/MIC ratios specifically in lactating goats for each specific pathogen to maximise the therapeutic success when doxycycline is given.

Finally, another important aspect of using a drug in an animal species is to know its safety and potential adverse effects. Doxycycline after IV injection caused tachycardia, high blood pressure, collapse and death in horses, while, in sheep and goats, it caused sialism, tachypnea, tremors and hind limb weakness [27,28,32]. Goats showed no local or systemic adverse effects after IV injection of doxycycline in the present study. Also, in previous studies after IM administration of this antibiotic in goats, symptoms of pain such as screaming, lying down, restlessness and swelling at the injection site have been reported [27]. In this study, all goats developed lameness, which resolved 24–48 h after antibiotic administration.

## 5. Conclusions

The IM formulation of doxycycline investigated in this study showed prolonged half-life, moderate tissue distribution, low bioavailability and caused lameness in all animals. Doxycycline rapidly crossed the blood–milk barrier, but exposure to the antimicrobial and the concentrations reached in milk were lower than those obtained in plasma. Although PK/PD ratios may be low with the pharmacokinetic data obtained with this formulation of doxycycline, at this dose and route of administration, doxycycline after IM administration could be useful for infections by moderate or highly susceptible bacteria in the mammary gland of goats. However, it may be necessary to test different doses of doxycycline or other routes of administration to achieve better surrogate markers and to establish repeated dosing regimens and clinical efficacy.

## Figures and Tables

**Table 1:** Validation parameters of the method for the analysis of doxycycline in the plasma and milk of goats using the HPLC conditions of the present study.

Source	Linear Regression Equation	Regression Coefficient	Precision (%)	Accuracy (%)	Recovery (%)
Plasma	y = 6.0 × 10^−7^x	R^2^ = 0.997	<6.9%	−6.0–10.9	80.7
Milk	y = 6.0 × 10^−7^x	R^2^ = 0.991	<8.7%	−4.8–10.5	82.3

**Table 2:** Pharmacokinetic parameters (mean ± SD) for doxycycline in plasma from dairy goats (*n* = 6) after parenteral administrations (intravenous (dose 5 mg/kg) and intramuscular (dose: 20 mg/kg)).

Parameters (Units)	Intravenous	Intramuscular
C_0_ (µg/mL)	15.75 ± 3.76	
λ_z_ (h^−1^)	0.32 ± 0.05	0.02 ± 0.01 ^a^
t_½λz_ (h) *	2.14	33.36 ^a^
V_z_ (L/kg)	0.85 ± 0.16	
V_ss_ (L/kg)	0.62 ± 0.08	
Cl (L/h/kg)	0.28 ± 0.08	
AUC_last_ (µg·h/mL)	18.78 ± 4.27	33.48 ± 11.14 ^a^
AUC_0→∞_ (µg·h/mL)	19.18 ± 4.90	43.96 ± 15.88 ^a^
%AUC_extrap_	2.06 ± 0.75	22.68 ± 5.28 ^a^
MRT (h)	2.32 ± 0.42	47.59 ± 7.43 ^a^
MAT (h)		45.26 ± 7.08
C_max_ (µg/mL)		1.99 ± 0.50
t_max_ (h)		3.2 ± 2.95
F (%)		45.60 ± 16.20

^a^ There are significant differences with the intravenous route (*p* < 0.05). * Harmonic mean. C_0_: concentration of the drug in the plasma immediately after intravenous administration; λ_z_: elimination rate constant; t_½λz_: half-life associated with the terminal slope (λ_z_) of a semilogarithmic concentration versus time curve; V_z_: apparent volume of distribution calculated according to the area method; V_ss_: apparent volume of distribution at steady state; Cl: total body clearance of the drug; AUC_last_: the area under the curve up to the last quantifiable point in time; AUC_0→∞_: the area under the plasma concentration versus time curve from zero to infinity; %AUCextrap: %AUC extrapolated; MRT: the mean residence time; MAT: the mean absorption time; C_max_: the peak plasma concentration after extravascular administration of the drug; t_max_: the time after extravascular administration to peak plasma concentration; F: the proportion of the administered dose that is available systemically (bioavailability).

**Table 3:** Pharmacokinetic parameters (mean ± SD) for doxycycline in milk from dairy goats (*n* = 6) after parenteral administrations (intravenous (dose 5 mg/kg) and intramuscular (dose: 20 mg/kg)).

Parameters (Units)	Intravenous	Intramuscular
λ_z_ (h^−1^)	0.33 ± 0.12	0.04 ± 0.03 ^a^
t_½λz_ (h) *	2.11	16.02 ^a^
MRT (h)	4.73 ± 0.46	36.08 ± 21.81 ^a^
AUC_last_ (µg·h/mL)	6.74 ± 4.26	17.61 ± 6.53 ^a^
AUC_0→∞_ (µg·h/mL)	7.28 ± 3.92	22.48 ± 9.09 ^a^
%AUC_extrap_	11.25 ± 9.32	20.25 ± 8.97 ^a^
AUC_milk_/AUC_plasma_	0.37 ± 0.12	0.51 ± 0.05 ^a^
t_max_ (h)	2.80 ± 1.10	5.00 ± 2.00
C_max_ (µg/mL)	1.38 ± 0.76	0.95 ± 0.28
C_max milk_/C_max plasma_	0.09 ± 0.05	0.49 ± 0.07 ^a^

^a^ There are significant differences with the intravenous route (*p* < 0.05). * Harmonic mean. For abbreviations, see footnote of Table 2.

## Data Availability

The data presented in this study are available on request from the corresponding author.
